# When headache is just too much—A case report and literature review of bilateral vertebral artery dissection

**DOI:** 10.1002/ccr3.8168

**Published:** 2023-11-12

**Authors:** Rakshaya Venu, Liene Muceniece, Neha Mittal, Roberto Mendoza, Christina Matl, Douglas Bettarelli

**Affiliations:** ^1^ Department of Internal Medicine Saveetha Medical College and Hospital Chennai India; ^2^ Department of Internal Medicine Texas Tech University Health Sciences Center Lubbock Texas USA

**Keywords:** case report, migraine headache, neck pain, neuroimaging, vertebral artery dissection, young female

## 1 INTRODUCTION

Spontaneous cervical artery dissection (sCAD) significantly contributes to strokes among individuals in their early and middle adulthood, accounting for approximately 25% of all ischemic strokes in patients aged 15–49 years.^1^ sCAD is categorized into two distinct types based on the specific artery involved: spontaneous internal carotid artery dissection (sICAD) or spontaneous vertebral artery dissection (sVAD).^1^, ^2^, ^3^ Spontaneous vertebral artery dissection (sVAD) is a less common, yet progressively acknowledged, contributor to strokes in individuals under 45 years of age. It represents approximately 2% of all ischemic strokes, with bilateral occurrences observed in 7% of cases. This condition is likely underestimated but potentially debilitating source of strokes among young adults.^4^ While both spontaneous internal carotid artery dissection (sICAD) and spontaneous vertebral artery dissection (sVAD) exhibit symptoms such as headache, neck pain, and delayed ischemic events, sICAD presents more commonly with focal neurological symptoms such as Horner syndrome.^5^

During its initial stages, sVAD is often misinterpreted as a musculoskeletal disorder; however, timely diagnosis is crucial in preventing severe disability or potentially life‐threatening strokes.^6^

We present a case of severe unilateral headache and neck pain with an initial diagnosis of migraine complicated with findings of bilateral vertebral artery dissection.

## 2 CASE REPORT

A 28‐year‐old female with a past medical history of migraine underwent initial evaluation at the local emergency room (ER) for a severe left‐sided headache that had lasted for the past 5 days. She described her pain as sharp and throbbing, radiating down to her neck and rated at 10/10 intensity. She had nausea, left‐eye blurred vision, along with painful eye movements (EOM) on the left, and dizziness. Patient denied any history of trauma. Cerebral computer tomography angiography (CTA) that was performed on the same day at the local ER reported fusiform aneurysmal dilation of the more distal right vertebral artery. Patient was immediately transferred to our hospital for neurosurgical evaluation.

**FIGURE 1:**
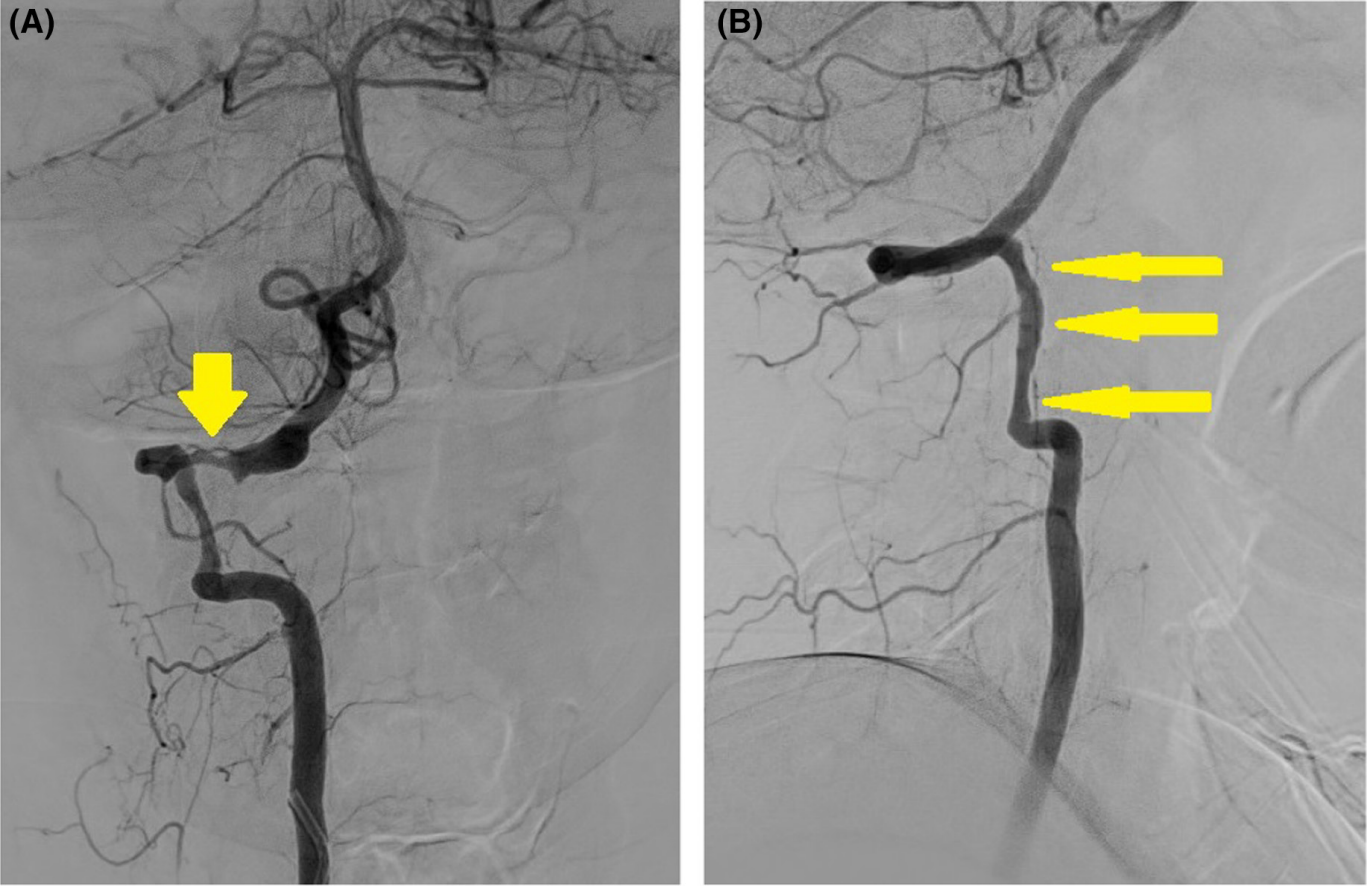
(A). Right vertebral dissection at C1 and C2 level just below the skull base. Yellow arrow: location of dissection. (B) The left vertebral at this level also shows significant irregularity and is suspected for a more subtle dissection as well and at the same levels of C1 and C2. Yellow arrows: location of suspected dissection.

On arrival, the patient was alert and in moderate distress and required repeated administration of antiemetics, non‐steroidal anti‐inflammatory drugs (NSAIDs), and opioids. On physical exam, no focal neurological or visual field deficits were observed. With an initial diagnosis of migraine with aura neurosurgery recommended a magnetic resonance imaging (MRI) of the brain. Requested imaging was performed as well as reported next day after patients transfer. Imaging revealed no restricted diffusion but was positive for stenosis of the right vertebral artery (VA) at the C1 and C2 levels, with an intramural hematoma on T1 and T2 sequences. Subsequently on the same day, the digital subtraction angiography (DSA) demonstrated bilateral vertebral artery dissection, with a more pronounced involvement on the right side (Figure 1). An intramural thrombus was also observed on the right side, raising questions about the underlying cause and the necessity for anticoagulation therapy. Interestingly, no true vertebral artery aneurysm, as suggested on prior CT imaging, was identified.

The patient denied taking any medications, or any family history of bleeding or clotting disorders, connective tissue disorders, or multiple sclerosis. All other common stroke risk factors like smoking, hypertension, oral contraceptive pills, and dyslipidemia were absent in this patient's case. Further investigation into the patient's history revealed no specific activity prior but that her neck pain had acutely started after lifting a crate of water bottles; however, migraine‐type headache started 5 days prior. Based on these findings, the patient was diagnosed with spontaneous bilateral artery dissection, likely secondary to lifting heavy weights, and concurrent left‐sided persistent migraines. The treatment plan involved initiating daily low‐dose aspirin (81 mg) and clopidogrel (75 mg), with a follow‐up CTA of the head and neck in 3 months to monitor the dissection and assess the resolution of the right vertebral mural thrombus.

This case highlights the diagnostic dilemma as well as the importance of considering rare disorders such as vertebral artery dissection in the differential diagnosis of persistent headaches in patients with migraine, particularly when associated with severe or refractory symptoms. Prompt recognition and appropriate management are crucial in preventing potentially devastating complications and improving patient outcomes. Although we could not definitely prove that bilateral vertebral artery dissection was the sole reason for her unilateral left‐sided headaches, it was a significant contributor to the patient's severity of symptoms.

## 3 DISCUSSION

Spontaneous cervical artery dissections, comprising carotid and vertebral dissections, account for approximately 20% of strokes in young adults. Among these, carotid artery dissections are more prevalent than vertebral artery dissections, occurring at a rate of approximately 1 in 100,000 individuals based on population studies.^7^ However, vertebral artery injury is a significant contributor to stroke and transient ischemic attack, particularly among younger patients. The preferred initial diagnostic imaging study is a CT angiogram, and treatment options vary depending on factors such as location, symptoms, and extent of the injury. Treatment may include fibrinolysis, anticoagulation, antiplatelet therapy, endovascular intervention, or surgical repair.^7^

Arterial dissection occurs when there are tears in the adventitia or intima, resulting in the formation of a hematoma within the arterial wall. This hematoma can narrow or block the vessel, obstructing blood flow.^8^ There are two perspectives regarding the main cause of this tear. The conventional belief is that a hematoma occurs when the inner layer tears, forming a false lumen that allows blood to penetrate the vessel wall. However, an alternative explanation suggests that the initial rupture happens within the wall's vasa vasorum, leading to the expansion of an intramural hematoma through the intimal layer and into the primary vessel lumen.^8^

Vertebral artery dissection can occur due to various causes, including blunt trauma, penetrating trauma, or spontaneous occurrences. Blunt trauma, particularly from motor vehicle crashes, is the most common cause, while falls, strangulation, and pedestrian accidents contribute less frequently. Even minor mechanical events preceding CAD or VAD as playing golf, visiting a hairdresser, can lead to vertebral artery injury. Penetrating trauma, such as from a gunshot wound, is rare but highly dangerous, often resulting in high mortality rates. Spontaneous injuries occur when the strength and structure of the artery wall are compromised. While some cases are associated with disorders affecting blood vessels or connective tissues, such as fibromuscular dysplasia, Ehlers‐Danlos syndrome, and Marfan syndrome, the majority of patients diagnosed with spontaneous vertebral artery dissection have no prior knowledge of any related underlying conditions. In most “spontaneous” cases, minor mechanical event coincides with the occurrence.^7^, ^9^, ^10^, ^11^

Studies conducted by Engelter et al. in 2013 and Traenka et al. in 2017 suggest that approximately 31%–40% of cervical artery dissections are preceded by some form of mechanical trauma. Although major incidents like roadside accidents and sports‐related injuries are linked to cervical artery dissection, more minor mechanisms are more commonly observed. Almost any type of mechanical events on neck, including activities like sneezing, nose‐blowing, heavy lifting, sudden head movements, falls, and fights, has been associated with cervical artery dissection.^7^, ^8^, ^12^

Diagnostic evaluation of cervicocerebral artery dissection involves various imaging techniques. Digital subtraction angiography (DSA) may indicate luminal stenosis or blockage, intimal flaps, or dissecting aneurysms or pseudoaneurysms. MRI can identify an intimal flap and the presence of a blood clot within the artery, and T1‐weighted images may reveal increased hyperintensity in the vessel wall. If both MRI and CT scan fails to detect any abnormalities, cerebral angiography may be necessary. While CT angiography (CTA) has a sensitivity of 100% and specificity of 98% in diagnosing vertebral artery dissections, conventional four‐vessel angiography remains the gold standard for diagnosis.^13^, ^14^

The management of cervical artery dissection aims to prevent stroke and future occurrences. There is currently no established treatment protocol or official guidelines for spontaneous cervical artery dissection. In most cases, the outlook is positive, with over 80% of patients experiencing spontaneous resolution within 3 months.^1^ Dual antiplatelet therapy is effective in preventing stroke and mortality among patients with symptomatic carotid and vertebral artery dissections within a three‐month timeframe. Anticoagulation carries an increased risk of intracerebral hemorrhage. However, if the dissection advances and causes hemodynamic instability, endovascular treatment may be necessary. Other indications for endovascular treatment include an enlarging pseudoaneurysm causing pain, cranial neuropathy, or recurrent stroke caused by distal embolization.^15^ While there are no established techniques to prevent neurological incidents or recurrence, avoidance of contact sports, neck manipulation, and sudden neck movements is recommended. Blood pressure regulation and avoiding medications containing estrogen are also important.^7^

Spontaneous vertebral artery dissection has a generally positive prognosis, with most patients experiencing a favorable outcome. However, a small percentage may experience significant neurological impairments, strokes, or even death. Many factors are associated with poor prognosis; for example, location—internal carotid artery dissection has worse outcomes than vertebral artery dissection. Other factors like the severity of associated ischemic stroke or sub arachnoid hemorrhage (SAH), advanced age, and higher stroke scores at the time of the diagnosis are all also associated with worse prognoses.^7^, ^16^

In conclusion, spontaneous vertebral artery dissection is less common but a significant contributor to strokes and transient ischemic attacks, particularly in young adults. The diagnosis involves various imaging techniques, and treatment options depend on factors such as location, symptoms, and extent of the injury. While most cases have a positive prognosis, a small percentage of patients may experience severe complications. Further research is needed to establish standardized treatment protocols and prevention strategies for spontaneous vertebral artery dissection.

## AUTHOR CONTRIBUTIONS

**Rakshaya Venu:** Conceptualization; writing – original draft; writing – review and editing. **Liene Muceniece:** Conceptualization; methodology; writing – original draft; writing – review and editing. **Neha Mittal:** Conceptualization; methodology; supervision; writing – review and editing. **Roberto Mendoza:** Conceptualization; investigation; writing – review and editing. **Christina Matl:** Investigation; writing – original draft. **Douglas Bettarelli:** Investigation.

## FUNDING INFORMATION

None.

## CONFLICT OF INTEREST STATEMENT

All authors have no conflict of interest.

## CONSENT

Verbal and written informed consent were obtained from the patient to publish this report in accordance with the journal's patient consent policy.

## Data Availability

This is a case report that utilizes references (links provided at the end of the report). Data sharing is not applicable to this article as no new data were created or analyzed in this study.
